# Fetal Organ Anomaly Classification Network for Identifying Organ Anomalies in Fetal MRI

**DOI:** 10.3389/frai.2022.832485

**Published:** 2022-03-18

**Authors:** Justin Lo, Adam Lim, Matthias W. Wagner, Birgit Ertl-Wagner, Dafna Sussman

**Affiliations:** ^1^Department of Electrical, Computer and Biomedical Engineering, Faculty of Engineering and Architectural Sciences, Ryerson University, Toronto, ON, Canada; ^2^Institute for Biomedical Engineering, Science and Technology (iBEST), a partnership between St. Michael's Hospital and Ryerson University, Toronto, ON, Canada; ^3^Division of Neuroradiology, The Hospital for Sick Children, Toronto, ON, Canada; ^4^Department of Medical Imaging, Faculty of Medicine, University of Toronto, Toronto, ON, Canada; ^5^Department of Obstetrics and Gynecology, Faculty of Medicine, University of Toronto, Toronto, ON, Canada

**Keywords:** deep learning, fetal MRI, Convolutional Neural Network (CNN), image classification, fetal disease, fetal organ anomaly

## Abstract

Rapid development in Magnetic Resonance Imaging (MRI) has played a key role in prenatal diagnosis over the last few years. Deep learning (DL) architectures can facilitate the process of anomaly detection and affected-organ classification, making diagnosis more accurate and observer-independent. We propose a novel DL image classification architecture, Fetal Organ Anomaly Classification Network (FOAC-Net), which uses squeeze-and-excitation (SE) and naïve inception (NI) modules to automatically identify anomalies in fetal organs. This architecture can identify normal fetal anatomy, as well as detect anomalies present in the (1) brain, (2) spinal cord, and (3) heart. In this retrospective study, we included fetal 3-dimensional (3D) SSFP sequences of 36 participants. We classified the images on a slice-by-slice basis. FOAC-Net achieved a classification accuracy of 85.06, 85.27, 89.29, and 82.20% when predicting brain anomalies, no anomalies (normal), spinal cord anomalies, and heart anomalies, respectively. In a comparison study, FOAC-Net outperformed other state-of-the-art classification architectures in terms of class-average F1 and accuracy. This work aims to develop a novel classification architecture identifying the affected organs in fetal MRI.

## Introduction

Fetal MRI is increasingly gaining importance for diagnosing fetal abnormalities detected on ultrasound. Accurate diagnosis is crucial for prompt diagnostic and therapeutic decision-making (Loomba et al., 2011).

A subset of machine learning called Deep Learning (DL) mimics how the human brain processes data, and thus, creates patterns that are used in decision-making. As data are fed through this mesh of neurons, each layer is responsible for processing a subset of the data and producing a result. Applications of DL include language processing, object recognition, speech recognition, segmentation, and classification. DL excels when learning from unstructured data. This makes DL a powerful tool in a clinical setting due to the high variability of morphology in biomedical images, especially in fetal imaging (Lundervold and Lundervold, 2019). Specifically, DL can be useful for medical fetal image classification, which attempts to assign a relevant diagnostic label to an image. Convolutional Neural Networks (CNN) are frequently used for classification tasks (Sarvamangala and Kulkarni, 2021) due to their high accuracy relative to other state-of-the-art architectures, and how computationally efficient they are (Alzubaidi et al., 2021). One of the main benefits of using CNNs is that they perform automatic feature extraction, avoiding the need for manual feature engineering. This makes CNN's very useful in research scenarios where features of interest are not well known or well understood, as in fetal image analysis.

Current fetal MRI classification algorithms generally focus on one organ of interest, most commonly, the fetal brain. This is likely due to the relatively high occurrence of fetal brain abnormalities, being three in one thousand fetuses (Boyd et al., 2011; Groen et al., 2017; Boyle et al., 2018), as well as the usual high image quality and frequency of fetal brain imaging. Recently, studies have differentiated between different brain anomalies by using attention mechanisms (Shi et al., 2020). However, their algorithm performed poorly at the detection of ventriculomegaly, with a relatively low classification accuracy of 67%. Another study classified brain anomalies while also using a dataset that included a wide range of gestational ages from 16 to 39 weeks (Attallah et al., 2019). The authors used 21 machine learning classifiers, specifically K-nearest neighbors, that obtained the highest classification accuracies and areas under the curve in the high 90s.

Existing algorithms that focus on organs other than the brain are often still organ-specific. Torrents-Barrena et al. reviewed popular segmentation and classification techniques for various fetal organs (Torrents-Barrena et al., 2019). They found that the placenta, brain, lungs, and liver benefit from feature extraction methods, and the heart benefits from intensity-based models. Organ-specific algorithms are limited in that they can only provide information or diagnoses regarding the target organ, thus ignoring any abnormalities in surrounding organs. However, since each organ has a unique appearance on medical imaging, creating a single algorithm that assesses all organs well can be challenging (Xie et al., 2020).

Nevertheless, having a single algorithm capable of assessing disorders that affect a variety of fetal organs would be desirable for efficient and holistic diagnoses. In recent years, more adaptive CNN methods have been proposed that can better detect the diverse characteristics of fetal organs and pathologies. A particularly useful development is the Squeeze and Excitation (SE) module proposed by Hu et al. In a standard CNN, the feature channels all have the same weights. On the other hand, SE modules implement adaptive weights to the channel-wise feature maps by modeling dependencies between convolutional channels (Hu et al., 2019). The SE module enables the use of global information by squeezing each channel through a global average pooling layer. A series of activation operations then produces a non-linear feature map that is concatenated with the original channels, resulting in an adaptive-weighted feature map. The result is a low-cost module that automatically emphasizes important features with global and local considerations (Rundo et al., 2019; Lo et al., 2021). Another new CNN methodology capable of multi-level feature analysis is the naive inception architecture (NI). NI modules improve classification accuracy by increasing network width and keeping network depth constant (Jin et al., 2019). They propose multiple filter sizes for the same network layer, allowing for multi-level feature analysis (Szegedy et al., 2014). While the NI module is capable of handling features of varying scales, the SE module can adapt to global and local intensity variations, making them both valuable tools in whole fetal analysis and diagnosis.

In our study, we take advantage of the benefits offered by the DL modules to develop a new architecture that can automatically and accurately identify affected fetal organs. We aim to develop a novel architecture by combining the previously established SE and NI modules to address the challenges of whole-fetal diagnosis. Our proposed architecture FOAC-Net aims to contribute to improvements in clinical settings by providing efficient and unbiased diagnoses critical for early lifesaving interventions.

## Materials and Methods

### Acquisition

This study was approved by the local research ethics board and the requirement for informed consent was waived due to the retrospective nature of the study.

Thirty-six de-identified whole-body fetal MRI datasets were included, as part of a collaborative study at The Hospital for Sick Children in Toronto, Canada. Datasets included a 3D SSFP sequence with SENSE along two dimensions (Seed and Macgowan, 2014) acquired on a 3T scanner. Coronal images were used as this orientation provided the most surface area for identifying fetal organs. Only one scan per patient was used if multiple images were available. Fetuses were excluded if they had a medical disease that had an occurrence of less than 1/100,000 for privacy concerns. Sequences with minor imaging artifacts were included if they contained minimal motion, chemical shift, or radiofrequency distortion, as assessed by two pediatric neuroradiologists. Gestational age at the time of image acquisition was between 20 and 37 gestational weeks (gw) with a distribution centered at 30 gw (30.62 ± 2.75 gw; mean ± std). Gestational age was evenly distributed across the training, validation, and testing datasets to avoid bias in a single dataset. Interpretation of the MRI was performed by radiologists.

### Dataset

Thirty-six T2-weighted coronal fetal MRI SSFP sequences were included in this study, each containing between 60 and 110 2D slices resulting in a total of 4,770 2D images. The data were divided into training, validation, and testing datasets in a 60/20/20 split. The resulting dataset had 2,904 slices in the training set and 933 slices in both the testing and validation sets. As our dataset was collected using different acquisition parameters, intensity normalization was used to enforce consistency and regularity in our dataset and avoid any biases induced by acquisition. The data was divided on a per-patient basis. For example, patients 1–22 are used in training, 23–28 in validation, and 29–36 in testing. Many biomedical applications typically divide the data on a per-patient basis (Khademi et al., 2021). The resolution of the images was of size 384 × 384. Fetal MRI anomalies were classified into four main cohorts: (1) brain abnormality, (2) cardiac abnormality, (3) healthy (no anomaly), and (4) spinal abnormality. The objective of this classification structure was to accurately classify prominent regions of fetal abnormalities present in MRI scans. Table 1 describes the number of slices per class, examples of diagnosis on MR imaging, and gestational age. A paired t-test was used to confirm that the differences in gw per anomaly were not statistically significant. The calculated *p* values for all classes were >0.05, using a paired t-test.

**Table 1:** Detailed evaluation of the fetal MRI dataset, training and validation, and examples of diagnoses.

**Class**	**Training**	**Validation**	**Testing**	**Examples of diagnosis**	**Gestational age (weeks)**
Brain	713	227	227	Hypoxic ischemic encephalopathy	30.41 ± 2.62
				Interhemispheric cyst	
				Agenesis of corpus callosum	
Heart	760	246	246	Intracardiac tuberous sclerosis	28.29 ± 2.52
				Central venous hypertension	
				Ventricular septal defect	
Spinal Cord	755	233	233	Spina bifida	31.61 ± 3.62
				Vertebra malformation	
				Cervical thoracic scoliosis	
Normal	676	227	227		32.17 ± 2.24
Total	2,904	933	933		

### CNN-Architecture Overview

**Figure 1:**
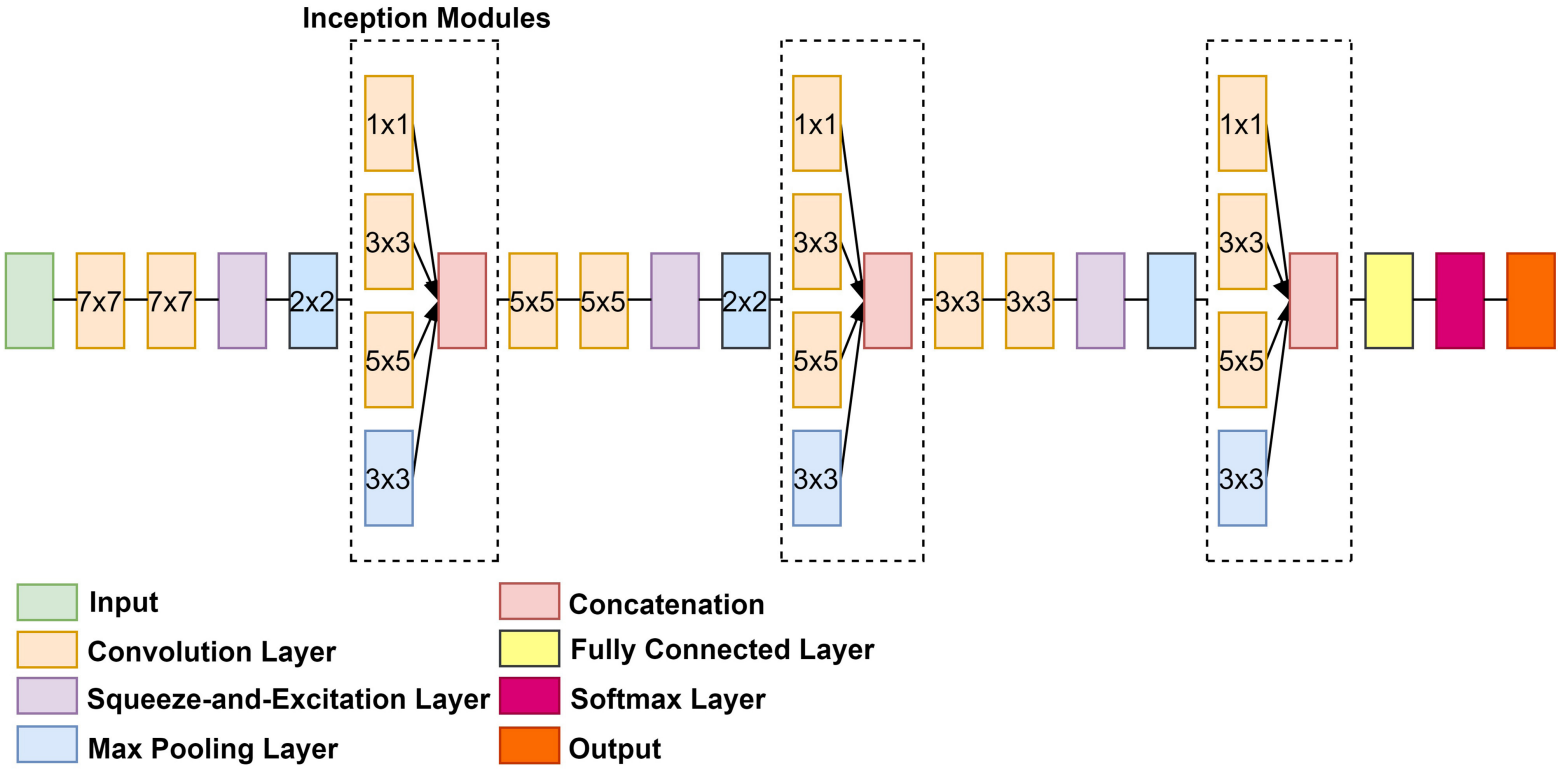
An overview of the proposed classification architecture, FOAC-Net.

The proposed architecture is illustrated in Figure 1. The architecture pathway consists of three SE modules applied after every convolution layer. Three NI modules were added after every maximum pooling operation. The excited channels from the SE modules were used as inputs to the NI modules. The NI modules then extracted multi-scale features by using convolutional filters of varying sizes. As a result, the proposed CNN selected relevant features that were emphasized for identifying the affected-organs classification task at hand while limiting the number of computational resources. Deeper along the data path, the kernel size of all filters was decreased in order to gradually focus on more refined features (Luo et al., 2017). This was done to force the network to consider global and local features that pertain to the affected organ.

### Squeeze-and-Excitation Module

**Figure 2:**
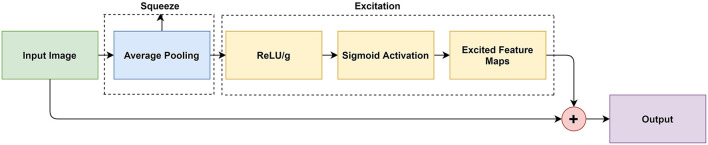
Block diagram representation of the SE module.

The SE modules were implemented after every convolution layer and were followed by the NI modules. This led to a rigorous feature selection process as the newly excited features from the SE modules were then processed in a multi-scale manner by the NI module. Figure 2 illustrates how the SE module was designed.

The SE modules were composed of two components: (1) the squeeze operation, (2) the excitation operation, and (3) the concatenation. In the squeeze operation, global average pooling was used to reduce the input to a single value across all channels. In the excitation operation, the model achieved the desired non-linearity. In the concatenation operation, the non-linear characteristics were applied to the original feature maps.

The squeezing operation is described by (1) where Un represents the feature map that is *squeezed* by Fsqueeze(), and the H and W are the dimensions of Un given by the height and width respectively. Global average pooling produces an average value representing each given feature map such that Zn represents the *n-th* element (Hu et al., 2019).


(1) $$\begin{array}{l} Z_{n}=\;F_{Squeeze}\left(U_{n}\right)=\;\frac{1}{H\times W\;}\overset{H}{\underset{i=1}{\sum }}\overset{W}{\underset{j=1}{\sum }}U_{n}\left(i,j\right) \end{array}$$

The excitation operation is described by (2). In order to model the interdependencies between the feature maps, a fully connected layer, ReLU layer, and a sigmoid activation layer are required. After the *squeezing* operation, each feature map was passed through a ReLU activation given as δ. This ReLU activation provided the model with the desired non-linearity. We divided the output by a constant value, g = 8. This will help the model with generalization and reduce the channel complexity. The resulting value was then applied to a sigmoid activation layer given as σ. The product is *n* excited feature maps that were ready to be used in upcoming layers (Hu et al., 2019).


(2) $$\begin{array}{l} s=\;F_{Excite}\left(Z_{n}\right)=\left(\;\sigma ,\;\left(\delta ,\frac{Z_{n}}{g}\right)\right) \end{array}$$

The concatenation operation is described in (3). In order to achieve the activations, we multiplied the excited feature maps by the original channels in the input image, where X = [ *x*_1_, *x*_2_, …, *x*_*n*_] were the channel wise concatenation between the excited feature maps *s*_*n*_ and the input feature map *U*_*n*_. This pipeline created adaptive weights for the feature channels for better spatial information (Hu et al., 2019).


(3) $$\begin{array}{l} \underline{X}=\;s_{n}u_{n} \end{array}$$

### Naïve Inception Module

**Figure 3:**
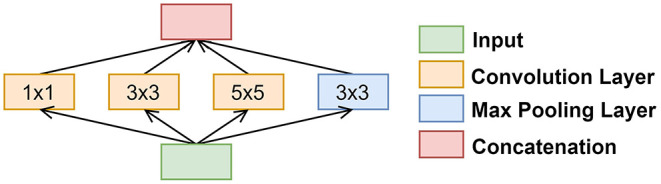
Block diagram of the naïve inception module.

The Naïve Inception modules were implemented after every maximum pooling layer that followed the SE module. There were several reasons for why we chose this specific inception module variant, namely (1) high-performance gain, (2) minimal increase in computational load when compared to other variants, (3) its ability to extract features from input data at varying sizes, and (4) utilization of 1 × 1 convolution filters. Figure 3 illustrates how we implemented the naïve inception module.

The 1 × 1 convolution behaves differently than its 3 × 3 and 5 × 5 convolutional counterparts. The 1 × 1 convolution (1) has dimensionality reduction of the input data in the module, (2) has a reduced number of filters, thus the output has a reduced number of channels relative to the initial input, and (3) can learn patterns across the channels of an image. The 3 × 3 and 5 × 5 convolution filters are used to extract spatial patterns at different scales. Traditionally, researchers would often have a tough choice in deciding what convolution filter size would be appropriate. Inception modules alleviate this issue by using different scales.

The maximum pooling layer, 3 × 3, and 5 × 5 convolution layers were padded to the dimensions of the input image. This allowed for all the convolution layers and maximum pooling layer to be concatenated. The concatenated output was then used as the input to the following convolution layers until the next naïve inception module was called once again.

### Experiments

The proposed method was trained and tested on an RTX 2070 Super graphics card. The proposed model was programmed in Python using Keras and TensorFlow and was trained using the Adam optimizer. The learning rate was set to 1e−4 and was scheduled to reduce to 1e−5 when the performance plateaued with a patience of 0. Categorical cross-entropy was used for our loss function. The size of the input images was 384 × 384 pixels. The batch size was set to 8, and 100 epochs were used for training. 5-fold validation was used during training to better utilize our data and improve generalizability. A basic set of image augmentation transformations were applied to the data. The basic transformations used included: shear, zoom, vertical and horizontal flip, width shift, and height shift.

Three metrics were implemented to evaluate the classification accuracy: (1) accuracy and (2) F1-Score. Accuracy is a metric that determines the proportion of true results among the total number of cases, which was calculated per 2D slice:


(4) $$\begin{array}{l} Accuracy=\;\frac{TP+TN}{TP+TN+FP+FN} \end{array}$$

Here *TP, TN, FP*, and *FN* represent the true positive, true negative, false positive, and false negative values, respectively. F1-Score is another classification metric that considers class imbalances, by considering the recall and precision. Recall considers what proportion of true positives are identified correctly. Precision considers what proportion of true identifications are identified correctly. F1-Score is the harmonic mean of the two and provides a new way of model interpretation.


(5) $$\begin{array}{l} Recall=\frac{TP}{TP+FN} \end{array}$$

(6) $$\begin{array}{l} Precision=\frac{TP}{TP+FP} \end{array}$$

(7) $$\begin{array}{l} F1\;Score=\frac{2\left(Recall^{*}Precision\right)}{Recall+Precision} \end{array}$$

Where the F1-Score is two times the product of the Recall and Precision scores, divided by the sum of Recall and Precision. Biomedical datasets often contain high variability and class imbalances; thus, the F1-Score provides a better representation of model performance as opposed to using solely accuracy. In the following tables, we present the average F1-Score over all classes.

We evaluate our own FOAC-Net as well as 3 other popular classification architectures including VGG-Net, ResNet, and DenseNet (Simonyan and Zisserman, 2014; He et al., 2015; Chollet, 2017; Howard et al., 2017; Huang et al., 2018; Jin et al., 2019). We chose these state-of-the-art architectures because they were demonstrated to work well in many imaging applications. These architectures follow the same hyper-parameter setup as previously described and were all implemented in Keras. We also conducted an ablation study to investigate the effects of the SE and NI components on the performance of FOAC-Net.

### Statistical Analysis

Architecture performance was evaluated using the class average of the previously mentioned metrics. Each model was trained 5 times and the reported performance is an average over the 5 trained versions. The significance of the performance differences between FOAC-Net and the other models were evaluated using independent t-tests on the accuracy and F1 scores. A 95% confidence interval and a significance level of *p* < 0.05 were used to determine significance. This statistical analysis is shown in Tables 2, 3.

**Table 2:** Mean testing results with standard deviation.

**Model**	**Accuracy (mean ± SD)**	**Accuracy *p* value**	**F1-score (mean ± SD)**	**F1-score *p* value**	**#of parameters**
FOAC-Net	85.32 ± 0.0025	–	85.24 ± 0.0024	–	10,336,178
VGG-16	78.93 ± 0.030	0.021	78.78 ± 0.028	0.016	138,357,544
VGG-19	82.43 ± 0.0079	0.011	82.83 ± 0.044	3.01e−05	143,667,240
ResNet-50	84.16 ± 0.0064	0.0048	79.56 ± 0.050	0.036	25,636,712
ResNet-101	82.56 ± 0.0016	1.87e−04	73.07 ± 0.041	0.0069	44,707,176
ResNet-152	81.93 ± 0.0082	0.0027	69.41 ± 0.0405	0.0025	60,419,944
DenseNet-121	82.04 ± 0.019	0.049	79.56 ± 0.033	0.042	8,062,504
DenseNet-169	83.82 ± 0.0060	0.020	82.05 ±0.018	0.039	14,307,880
DenseNet-201	84.35 ± 0.0012	0.0099	77.59 ± 0.0093	1.56e−04	20,242,984
Inception-V3	75.75 ± 0.037	0.011	72.43 ± 0.033	3.01e−05	23,851,784
Inception-ResNet	82.02 ± 0.019	0.043	71.99 ± 0.0026	2.82e−07	55,873,736
Mobile-Net	80.76 ± 0.012	0.0033	71.38 ± 0.016	1.11e−04	4,253,864
Xception	83.65 ± 0.0091	0.0417	78.58 ± 0.029	0.016	22,910,480

**Table 3:** Ablation study with mean standard deviation.

**Model**	**Accuracy (mean ± SD)**	**Accuracy *p* value**	**F1-score (mean ± SD)**	**F1-score *p* value**
FOAC-Net	85.29 ± 0.0025	–	85.24 ± 0.0024	–
Proposed-NI	82.01 ± 0.0018	1.01e−04	82.11 ± 0.0021	4.98e−05
Proposed-SE	80.85 ± 0.0028	5.30e−05	80.82 ± 0.0223	1.76e−05
Proposed-NI-SE	80.74 ± 0.0048	1.63e−04	80.82 ± 0.0047	1.10e−04
Proposed (K)	79.69 ± 0.0075	2.84e−04	79.69 ± 0.0074	2.40e−04
Proposed (K)-NI	79.49 ± 0.0093	5.97e−04	81.56 ± 0.019	0.029
Proposed (K)-SE	79.25 ± 0.0097	4.44e−04	79.66 ± 0.034	0.046
Proposed (K)-SE-NI	78.98 ± 0.0064	2.52e−04	79.52 ± 0.039	0.033

## Results

FOAC-Net achieved the highest classification accuracy and F1-score. The other models that were tested on had classification accuracies in the high seventies to low eighties. However, the F1-score for some models was in the high sixties to low seventies. This is illustrated in Table 2.

Table 3 presents the results of the ablation study that was conducted on our proposed FOAC-Net. We started with the proposed architecture and evaluated the performance of the model by removing important aspects such as NI and SE modules. In Table 3, we show the difference value between the performance of the ablated model and the proposed FOAC-Net. We also evaluated the performance of the model using 3 × 3 kernel sizes (denoted by k) across the entire model as opposed to our descending approach.

**Figure 4:**
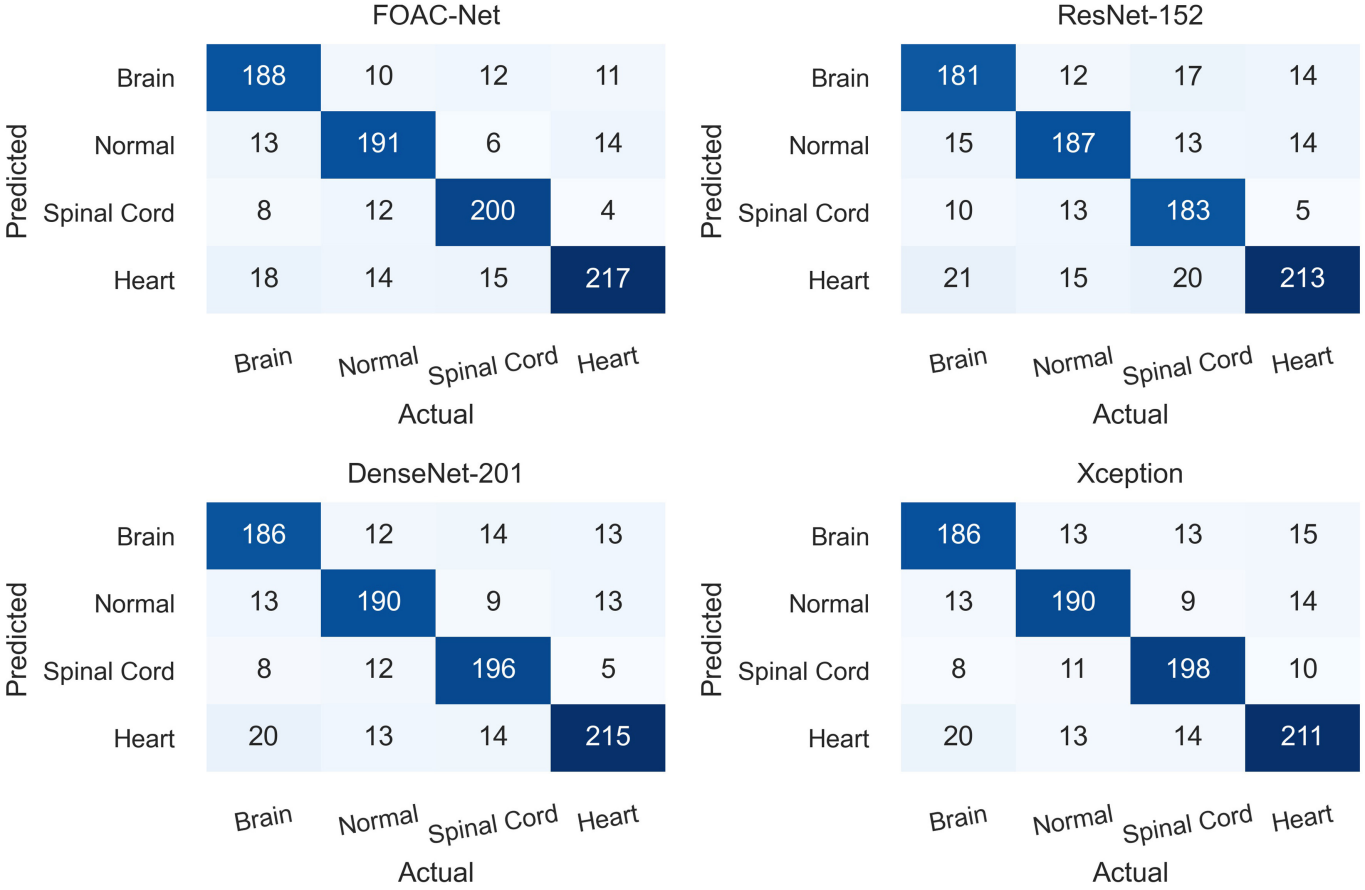
Confusion matrix evaluating specific class performance on the proposed architecture.

Figure 4 illustrates the class-by-class performance of the FOAC-Net architecture in a confusion matrix. Each class incorrectly predicted another class at least once. However, for each class, the precision, recall, and accuracy were all over 80%. FOAC-Net performed the worst when classifying the heart at 82.20%. FOAC-Net performed the best when classifying spinal cord anomalies at 89.29%. Each box in the confusion matrix represents what FOAC-Net predicted. For example, in the first box (top-left-most box), FOAC-Net correctly predicted the brain on 188 occurrences. The diagonal row represents correct classifications or true positive values. The recall can be calculated by taking the true positive class in the given column and dividing it by the sum of the entire column. Similarly, we can obtain the precision value by taking the true positive class in the given row and dividing it by the sum of the entire row.

## Discussion

Our study presents a novel CNN architecture, FOAC-Net, which can achieve accurate 2D fetal organ anomaly classification when trained on second and third-trimester images. FOAC-Net outperformed other state-of-the-art classification architectures. Specifically, FOAC-Net obtained the highest accuracy (85.32%) relative to 12 other prevalent architectures. In addition, FOAC-Net also attained the highest F1-score at 85.24% indicating that the model has low incidences of false negatives, which is crucial for medical classification tasks. These results showed that a combination of SE and NI modules had a positive effect on classification performance. The performance presented by FOAC-Net has significant implications regarding the potential for DL to improve antenatal diagnosis of fetal anomalies in the clinic.

State-of-the-art classification architectures including the Res-Net, Dense-Net, and VGG variants, InceptionNet, MobileNet, and Xception all performed worse compared to the proposed model. VGG-16 and 19 performed worse than our proposed model, but unlike the other models, there was very little difference between the accuracy and F1 scores of the VGG models. The Res-Net models had better performance than the VGG models but still performed worse than the proposed model. The accuracies ranged from 81 to 84; however, the F1 scores were generally low. This is likely due to the models' inability to differentiate between relevant anatomy. The added model depth from Res-Net architecture likely resulted in increased complexity, leading to overfitting and the inability to differentiate anatomy. The Dense-Net variants also obtained high accuracies while maintaining a high F1score, unlike the Res-Net series, but Dense modules were still unable to compete with FOAC-Net. The inception-based models performed generally worse than the previously described models. Mobile-Net and Xception had accuracies ranging from 80 to 83%, but lower F1-score performance in the high 70 s. The improved performance of our model relative to the competitors was found to be significant, with *p* values on the F1 and accuracy scores being <0.05 as shown in Tables 2, 3. It can be seen from Figure 4 that other state-of-the-art classification models perform slightly worse on a class-by-class basis when compared to FOAC-Net.

It can be seen from Table 2 the number of parameters given for each state-of-the-art classification model. Our proposed FOAC-Net has 10.36 million parameters which are higher than DenseNet-121 and Mobile-Net. However, both classification architectures are out-performed by FOAC-Net by 3.32 and 4.56% respectively. The small increase in parameters justifies the performance increase. FOAC-Net only took another few minutes to train. When observing classification models with a larger number of parameters such as VGG-16, ResNet-152, Xception, and more, we did not see an increase in classification accuracy or F1 score. FOAC-Net offers high classification performance while minimizing the number of parameters needed for training.

We conducted our ablation study (outlined in Table 3) to determine which individual components were responsible for the relative success of FOAC-Net. The first experiment demonstrated that the removal of NI modules lowered the accuracy and F1-score. Similarly, removing SE modules also diminished the performance of the model even more than NI modules. However, changing the kernel sizes to 3 × 3 across the entire architecture lowered the performance the most. This demonstrates that the larger kernel sizes at the start of the model were able to extract larger and more general features better, which can translate into higher performance. After changing the kernel sizes and removing either NI or SE, the performance significantly decreased. This ablation study demonstrated that the performance of the model was impacted by the following (from greatest to least): (1) kernel size, (2) SE modules, and (3) NI modules. Embedding SE modules in FOAC-Net seemed to improve classification performance, however, when paired with the NI modules the performance was increased even further. We believe the NI modules took advantage of the adaptive weights produced by the SE modules and were able to learn well from them. The opposite also holds true: NI modules do not perform as well on their own compared to when they are paired with SE modules. These deeper calculations allowed for high performance. Overall, all three components contributed to the success of our network.

Figure 4 shows the performance of FOAC-Net on each target class in the form of a confusion matrix. The precision score for the cardiac class was 82.20%. This relatively low value is likely attributed to the heart's small size and motion. For the brain class, the model achieved moderate precision scores of around 85%. However, the brain class also had a low recall score, indicating high false-negative classifications. It is possible that the relatively large number of congenital anomalies in the fetal brain was the reason for the lower performance. The large variance in anatomical abnormalities in the brain makes training difficult and thus contributes to the relatively low recall of brain abnormalities. The normal class and spinal cord class obtained a precision score of 84.14 and 85.83% and a recall score of 85.27 and 89.29% respectively. Both the normal and spinal cord classes out-perform the brain and heart class, which is likely due to relative anatomical simplicity when compared to the brain and heart.

**Figure 5:**
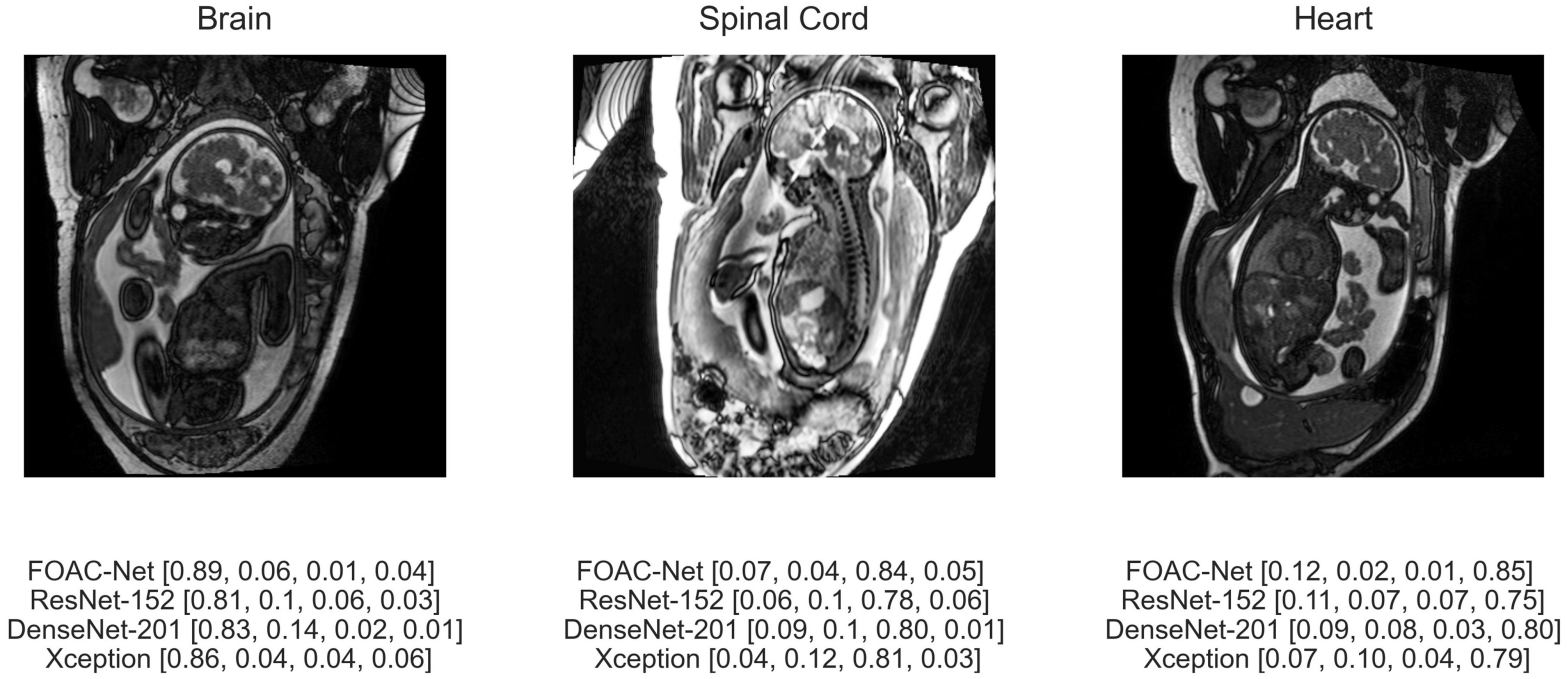
Predicted classification output of FOAC-Net, ResNet-152, DenseNet-201, and Xception (normalized prediction probabilities). Target classes are [brain, normal, spinal cord, heart], respectively.

Figure 5 illustrates the predicted classification results of FOAC-Net on a patient. Quantitatively, the fetal brain is the most prominent organ in the image The algorithm predicted that this image has an 89% chance of showing a brain anomaly, 6% chance for normal anatomy, 1% for spinal cord abnormalities, and 4% for cardiac abnormalities. The radiologist report determined this fetus to have mild left ventriculomegaly, therefore FOAC-Net was able to predict the image as one containing a brain abnormality correctly. We can see that FOAC-Net has better classification performance compared to other state-of-the-art classification models on all affected organs. However, the normalized probabilities show that the other classification models can still identify the affected organ but with lower accuracy.

Our model was effective in identifying the affected organ. Despite the relatively high performance of FOAC-Net, we believe the performance is likely to increase. Fetal MR imaging datasets suffer from high anatomical and positional variability, making the small number of patients in our dataset a primary factor in limiting the performance of our architecture. Expanding the dataset, and thus expanding the anatomical orientations of the dataset would likely improve classification performance. Having more fetal data with varying orientations would provide the proposed model with more diversity and a deeper understanding of the appearance of fetal disorders. When implementing a 2D slice-by-slice approach the heart was often difficult to identify.

## Conclusion

We present an automated whole fetal MRI affected-organ classification method using DL techniques. FOAC-Net is a novel architecture that utilizes the combined effects of SE and NI modules to extract excited features at varying scales to improve classification performance. FOAC-Net showed promising results, while also offering improvements over other state-of-the-art architectures. The proposed model provides an accurate prediction of the classes at 85.32% accuracy. The proposed architecture was successful in fetal MRI affected-organ classification, suggesting that automated classification techniques can be useful to increase efficiency and accuracy in clinical settings.

## Data Availability Statement

The datasets presented in this article are not readily available because the collaborating hospital did not approve their release to the general public. Requests to access the datasets should be directed to dafna.sussman@ryerson.ca.

## Ethics Statement

The studies involving human participants were reviewed and approved by Hospital for Sick Children and Ryerson University. Written informed consent for participation was not required for this study in accordance with the national legislation and the institutional requirements.

## Author Contributions

JL and DS designed the proposed method. JL and AL performed experiments. JL, AL, and DS wrote the manuscript. JL, MW, and BE-W obtained clinical data. All authors discussed the results, reviewed the final version of the manuscript, contributed to the article, and approved the submitted version.

## Funding

Ryerson University, the Faculty of Engineering and Architectural Sciences, and NSERC-Discovery Grant (RGPIN-2018-04155) (DS) provided funding for the study.

## Conflict of Interest

The authors declare that the research was conducted in the absence of any commercial or financial relationships that could be construed as a potential conflict of interest.

## Publisher's Note

All claims expressed in this article are solely those of the authors and do not necessarily represent those of their affiliated organizations, or those of the publisher, the editors and the reviewers. Any product that may be evaluated in this article, or claim that may be made by its manufacturer, is not guaranteed or endorsed by the publisher.
